# Batch preparation of nanofibers containing nanoparticles by an electrospinning device with multiple air inlets

**DOI:** 10.3762/bjnano.14.15

**Published:** 2023-01-23

**Authors:** Dong Wei, Chengwei Ye, Adnan Ahmed, Lan Xu

**Affiliations:** 1 College of Textile and Engineering, Soochow University, Suzhou 215123, China

**Keywords:** batch preparation, electric field simulation, electrospinning device, functional nanofibers, nanoparticles

## Abstract

With the increasing application of electrospun nanofibers, the batch preparation of high-performance functional nanofibers containing nanoparticles has become a research hotspot. As the distribution uniformity of nanoparticles in functional nanofibers has a great impact on their performance, an electrospinning device with multiple air inlets, which has a copper porous spinneret, is proposed to obtain functional nanofibers with higher yield and more uniform distribution of nanoparticles. The mechanism of batch preparation of functional nanofibers containing ZnO nanoparticles by the device was studied through experiments and theoretical analysis. The experimental data are in good agreement with the theoretical analysis results, which showed that under the appropriate voltage (50 kV) and air flow (50 m^3^/h), the device could keep ZnO nanoparticles contained in the spinning solution evenly dispersed during the spinning process, thus obtaining functional nanofibers with more uniform distribution of ZnO nanoparticles, whose quality and yield were higher than those prepared by other high-yield electrospinning devices.

## Introduction

In recent years, due to the characteristics of high specific surface area, good electrical conductivity, stable physical and chemical properties, as well as fast charge and discharge, the application of electrospun nanofiber-based materials in supercapacitor electrode materials has attracted great attention [1–2]. Although traditional single-needle electrospinning (SNE) devices are widely used and simple to operate, their industrialization is limited due to the extremely low output [3]. Compared to SNE, multineedle electrospinning improves the output to a certain extent, but the mutual interference of electric fields between needles affects the spinning process, and the needle blockage problem has not been solved [4]. Needle-free electrospinning devices fundamentally solve the needle blockage problem and can prepare micro/nanofibers more effectively [5].

With the expanding application field of electrospun nanofibers and the change of human needs, various functional nanofibers have emerged as promising materials, such as conductive fibers [6], phase change fibers [7], antistatic fibers [8], and antibacterial fibers [9]. Therefore, the batch preparation of high-performance functional nanofibers by electrospinning has become a current research hotpot [10]. The properties of spinning solutions used to prepare different functional nanofibers are various. When the spinning solution contains insoluble functional nanoparticles, these particles are usually not well dispersed in the solution. Especially in the spinning process, when the solution stays in the reservoir, the nanoparticles will sink down. This results in an obvious stratification of the solution and an uneven distribution of the functional nanoparticles in the final products, reducing the performance of products [11–14].

**Figure 1:**
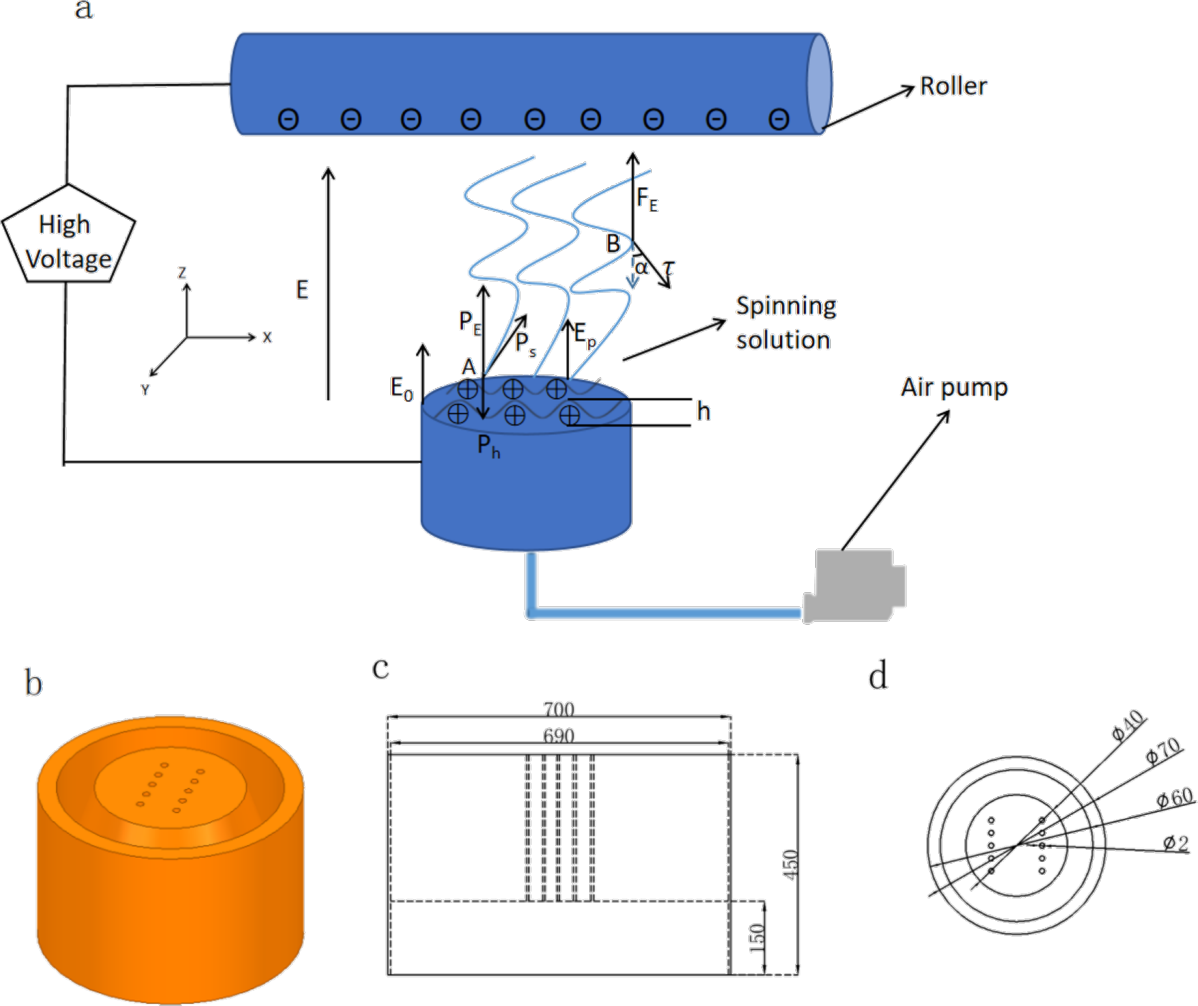
Diagram of EMAI (a); 3D diagram of the copper porous spinneret (b) and its corresponding longitudinal section (c) as well as cross section (d).

In our previous works, some self-made free surface electrospinning (FSE) devices were proposed for high-throughput fabrication of nanofibers [15]. Especially, the spherical section FSE (SSFES) device developed by our research group can be used to prepare nanofibers with higher yield and quality compared with other works [16]. However, in the SSFES device, the nanoparticles in the spinning solution easily sink down, resulting in an uneven distribution of nanoparticles in the obtained fibers [17]. Due to their stable physical and chemical properties, good biocompatibility, excellent photoelectric properties, non-toxicity, strong antibacterial activity and low price, ZnO nanoparticles can be used in various fields to improve the performance and technical indicators of products [17–20]. An electrospinning device with multiple air inlets (EMAI) was designed and developed in this paper to prepare functional nanofibers containing ZnO nanoparticles in batches, as shown in Figure 1. The device enabled the ZnO nanoparticles contained in the spinning solution to maintain uniform dispersion in the batch preparation process of nanofibers by means of air flow produced through multiple pores. The airflow reduces the agglomeration of nanoparticles, thus yielding nanofibers with uniform ZnO loading. In addition, experiments and theoretical analysis were used to discuss the spinning mechanism of this new device for the batch preparation of nanofibers, and the optimal spinning parameters were determined.

## Materials and Methods

### Materials

Polyacrylonitrile (PAN, average molecular weight 15000) and zinc oxide nanoparticles (ZnO, purity 99.7%, spherical particles with an average diameter of 50 nm) were supplied by Shanghai Sinopharm Chemical Reagent Co., LTD. *N*,*N*-Dimethylformamide (DMF) was purchased from Shanghai Lingfeng Chemical Reagent Co., LTD. All materials are analytical grade and can be used without purification.

### Preparation of spinning solution

Firstly, 4.5 g PAN and 44 g DMF solution were weighed separately by an electronic balance (XJ120A, Shanghai Precisa Co., LTD), mixed and placed on an unheated magnetic stirrer (HJ-6A, Gongyi Yuhua Instrument Co., LTD.) for stirring for 24 h. Then 1.5 g ZnO nanoparticles were added into PAN solution, where the mass ratio of PAN to ZnO was 3:1, and the mixture was stirred for 4 h to obtain the homogeneous ZnO/PAN spinning solution. The mass fraction of PAN to spinning solution was 9 wt %, and that of ZnO to spinning solution was 3 wt %.

### EMAI experiments

All EMAI experiments were performed at 25 °C and 60% relative humidity. The following MEAI parameters were used. The receiving distance was 18 cm, the speed of the receiving drum was 300 r/min, the air flow rates were 0, 50, 100, and 150 m^3^/h, and the spinning voltages were 40, 50, and 60 kV.

### Measurement and characterization

The spinning solution viscosity was measured by a viscosity instrument (SNB-1, Shanghai Ruifang Co., LTD.). The spinning solution conductivity was tested by a conductivity tester (DDS-307A, Shanghai INESA Analytical Instrument Co., LTD.). The yield of nanofibers fabricated by EMAI was determined using an electronic balance (XJ120A, Precisa LTD.). The nanofiber morphology was investigated by a scanning electron microscopy (SEM, Hitachi S4800, Hitachi LTD.), and Image J software (National Institute of Mental Health) was used to characterize the fiber diameter distribution by random selection of 100 nanofibers from ten SEM images of each sample. Simultaneously, the element distribution on the sample surface was characterized by a desktop SEM (TM3030, Hitachi LTD.).

### Electric field simulation

Maxwell 3D was used to simulate the electric field distribution of EMAI under different voltages (40, 50, and 60 kV). A simplified 3D model of the porous copper spinneret with a diameter of 70 mm and a height of 45 mm is shown in Figure 1b. Ten holes with a diameter of 2 mm were drilled into the spinneret. The specific simulation parameters were as follows: The conductivity of copper was 5.8 × 10^11^ µS/cm, the viscosity of the spinning solution was 377 mPa·s, and the conductivity of the spinning solution was 797 µS/cm [21].

## Results and Discussion

### EMAI process and batch preparation of nanofibers

**Figure 2:**
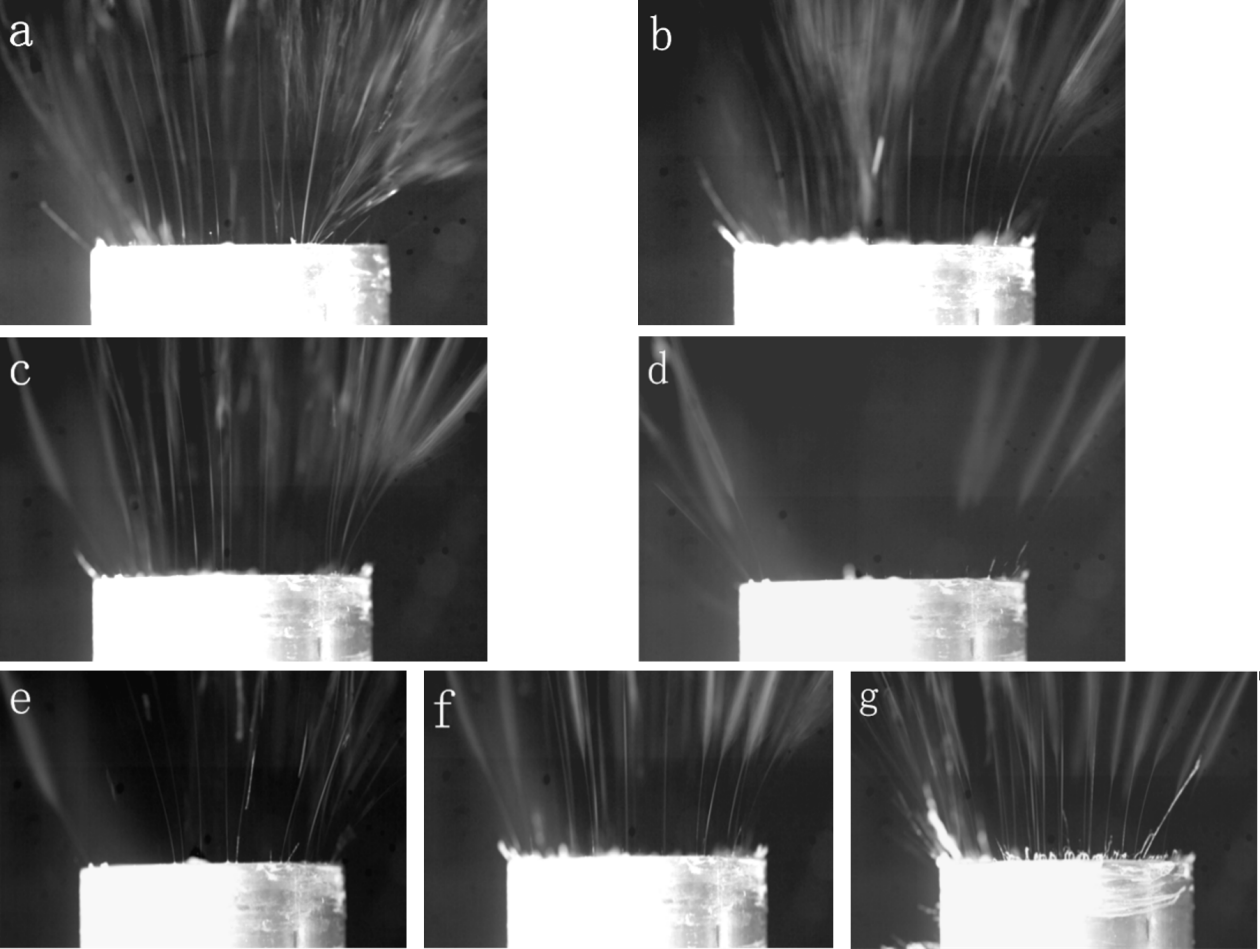
EMAI spinning processes at different air flow rates (150 m^3^/h (a), 100 m^3^/h (b), 50 m^3^/h (c), and 0 m^3^/h (d)) and voltages (40 kV (e), 50 kV (f), and 60 kV (g)).

Figure 2 shows that, in the EMAI process, most of the jets were ejected from the edge of the spinneret, the edge of the groove, and the air inlets. This was because the edge part would produce higher electric field intensity due to the tip effect, while the air inlets would produce more jets due to the auxiliary effect of air flow. When the air flow rate was 150 m^3^/h (Figure 2a), because of the excessive air flow, a large number of bubbles were generated on the solution surface, and many droplets appeared on the receiving drum, which greatly affected the spinning effect. When the air flow rate was 100 m^3^/h (Figure 2b), there were still many bubbles on the solution surface and some droplets on the receiving drum, illustrating that the air flow was still too large. When the air flow rate was 50 m^3^/h (Figure 2c), the whole solution surface became convex, and no obvious droplets appeared between the nanofibers on the receiving drum. When there was no air flow (0 m^3^/h) (Figure 2d), the number of jets obviously decreased and almost all of them were concentrated at the edge of the spinneret. Therefore, the air flow rate of 50 m^3^/h was more conducive to spinning.

Based on the above results, the spinning effects of this device under different applied voltages at the optimal air flow of 50 m^3^/h were studied. It was found that, when the applied voltage was 40 kV (Figure 2e), less jet formation and low spinning efficiency were achieved. When the voltage was 60 kV (Figure 2f), more but unstable jets were formed due to too high electric field intensity, which would result in coarser fibers and fiber bundles. Therefore, the optimal applied voltage value was 50 kV (Figure 2g), due to the stable jets generated under this voltage and the obtained fibers with smaller diameter and more uniform particle distribution.

Figure 1a shows the force analysis of point A on the free surface of spinning solution and point B on the jet formed during the EMAI spinning process when ignoring air resistance and environmental interference. The spinning solution was assumed to be an incompressible ideal fluid [22]. According to previous studies [23], under a high voltage electric field, in point A there is the joint action of mass force (*P*_h_) caused by the fluctuation height of spinning solution, surface tension (*P*_s_) of the spinning solution, and electric field force (*P*_E_) produced by the applied voltage. These forces determine whether a jet could be formed at point A. The polymer fluid surface tension would make the liquid surface shrink and bend as much as possible, and the change of external electric field would interact with the accumulated charges on the polymer fluid surface, making the charge density on the charged fluid surface uneven. Accordingly, the formulas to calculate these three forces are as follows:


[1] $$P_{\text{h}}=\rho gh$$

[2] $$P_{\text{s}}=-\gamma \left\{\frac{\delta^{2}h}{\delta x^{2}}\right\}$$

[3] $$P_{\text{E}}=-\frac{1}{2}\varepsilon_{0}E_{0}^{2}-\frac{1}{2}\varepsilon_{0}\varepsilon_{\alpha }\left(\varepsilon_{\alpha }-1\right)\cdot E_{\text{p}}kh$$

where ρ is the density of spinning solution (kg/m^3^), *g* is the gravitational acceleration (m/s^2^), *h* is the fluctuation height of the polymer spinning solution (m), γ is the surface tension coefficient of the spinning solution (N/m), ε_0_ is vacuum dielectric constant, *E*_0_ is the edge electric field intensity (V/m), *E*_p_ is the electric field intensity of the thin liquid surface (V/m), ε_α_ is the dielectric constant of the polymer, and *k* is the amount of radial fluctuations on the spinning solution surface.

In addition, the centripetal force *F*_1_ at point B is generated by the horizontal component of the viscous force (τ), which could weaken the instability of the jets. The resultant vertical upward force *F*_2_ generated by the applied electric field force *F*_E_ and the vertical component of viscous force would push the jets upward. The formulas of the resultant forces (*F*_1_ and *F*_2_) could be written as follows [24]:


[4] $$F_{1}=\tau \sin\alpha ,$$

[5] $$F_{2}=F_{E}-\tau \cos\alpha =q_{\text{B}}E_{\text{B}}-\tau \cos\alpha ,$$

where α is the angle between the viscous force and the vertical direction, *E*_B_ is the electric field intensity at point B, and *q*_B_ is the charge determined by the spinning solution properties at point B, which is generally an integer multiple of the primary charge (1.60 × 10^−19^ C).

On the basis of the spinning effects under different voltages (Figure 2e–g) and force analysis, it could be found that the distribution of the electric field intensity plays a very important role in the EMAI process for producing more and stable jets on the whole spinning surface. In the following, the nanofibers fabricated by EMAI are characterized and the electric field distribution in the EMAI process is simulated to illustrate the influences of electric field distribution on the spinning process and nanofiber quality.

### Characterization of nanofibers

**Figure 3:**
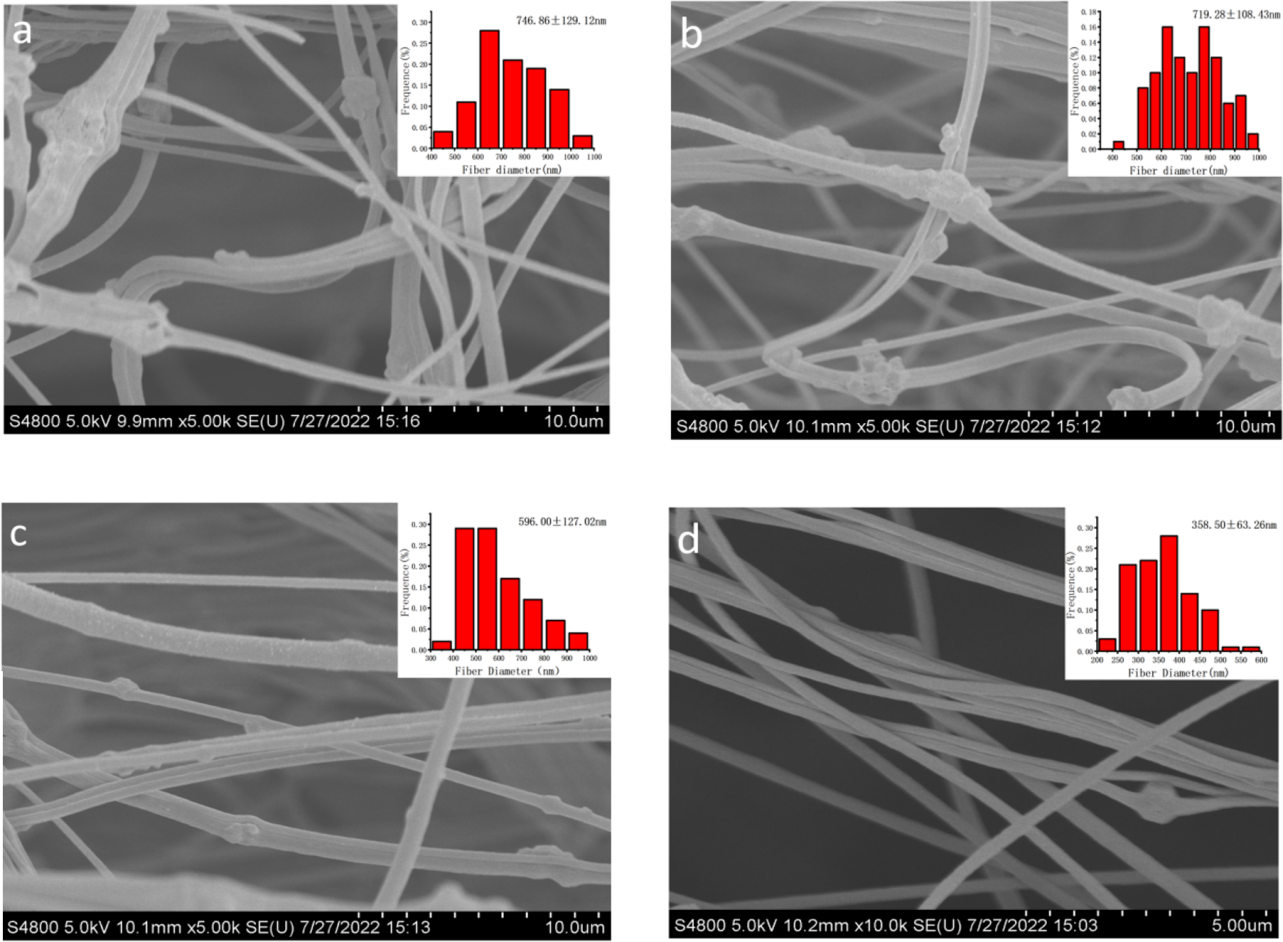
Morphology and corresponding diameter distribution of nanofibers obtained by EMAI at different air flow rates (150 m^3^/h (a), 100 m^3^/h (b), 50 m^3^/h (c), and 0 m^3^/h (d)).

Figure 3a–d shows the morphology and the corresponding diameter distribution of nanofibers obtained at different air flow rates. When the air flow rate was 150 m^3^/h (Figure 3a), due to the excessive air flow, the fibers easily adhered to each other, making the average diameter of the fibers larger (746.86 ± 129.12 nm) and leading to serious agglomeration of nanoparticles in the fibers. When the air flow rate was 100 m^3^/h (Figure 3b), the adhesion between fibers was weakened, resulting in the decrease of average fiber diameter (719.28 ± 108.43 nm) and a reduction of nanoparticle agglomeration in the fibers. When the air flow rate was 50 m^3^/h (Figure 3c), there was almost no adhesion between fibers. The average diameter of fibers was smaller (596 ± 127.02 nm), and the nanoparticles in the fibers were not agglomerated and evenly distributed. When there was no air flow (Figure 3d), the particles in the fibers were not only very few, but also agglomerated. This might be due to the sinking down of the nanoparticles in the spinning solution without the assistance of air flow. Therefore, the morphology of the fibers obtained using an air flow rate of 50 m^3^/h was the best, in which the nanoparticles were evenly distributed without agglomeration. This result is consistent with the analysis result in Figure 2a–d.

**Figure 4:**
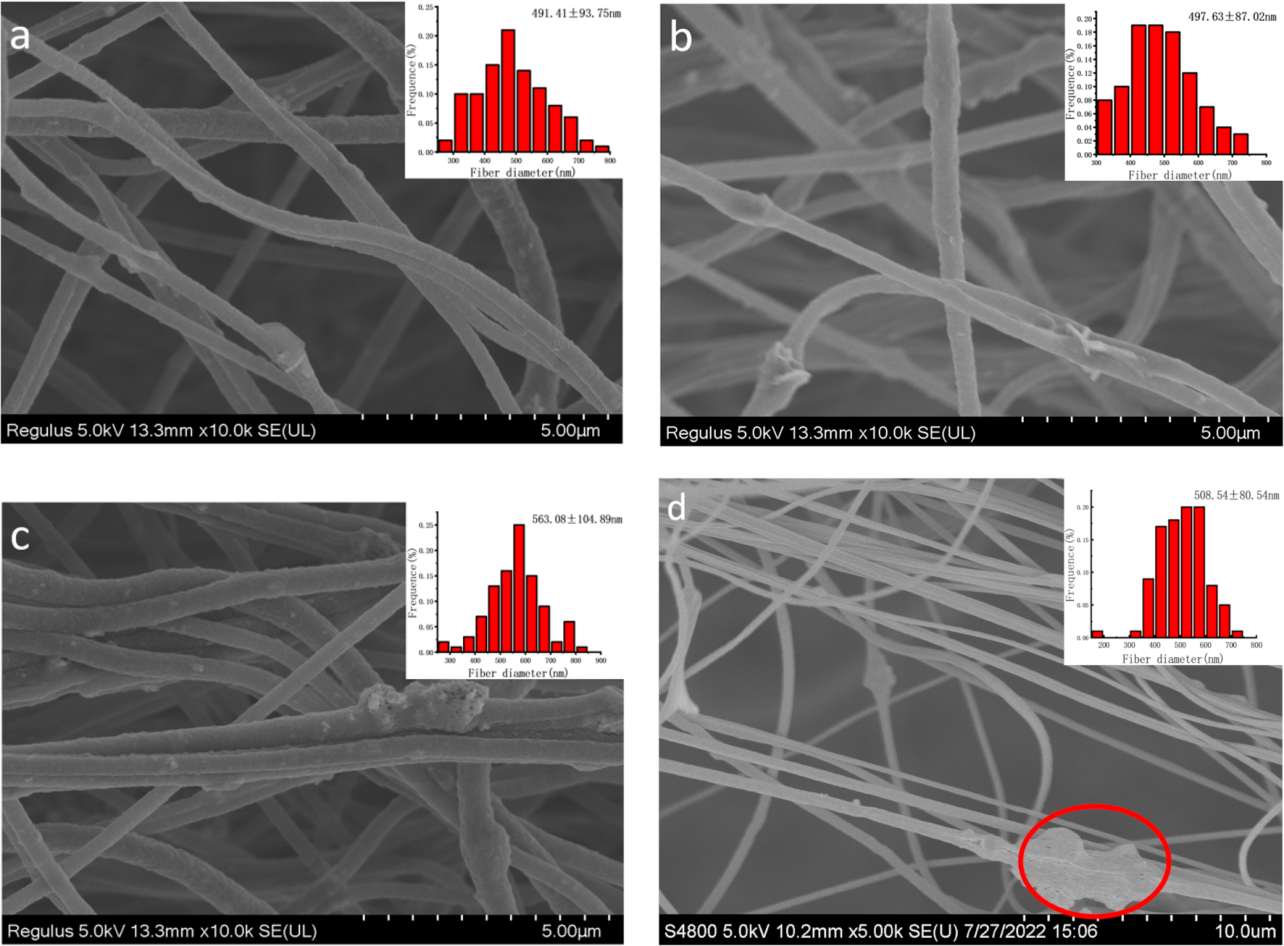
Morphology and corresponding diameter distribution of nanofibers obtained by EMAI at different voltages (40 kV (a), 50 kV (b), and 60 kV (c)) and by SSFSE (d).

In addition, the morphology and corresponding diameter distribution of nanofibers produced at different applied voltages with the optimal air flow rate of 50 m^3^/h were investigated. As shown in Figure 4a, when the spinning voltage was 40 kV, the electrospun nanofibers had fewer particles, and the average nanofiber diameter was 491.41 ± 93.75 nm. When the spinning voltage was 50 kV (Figure 4b), the nanoparticles in the nanofibers were not only abundant but also evenly distributed, and the average nanofiber diameter was the smallest (497.63 ± 87.02 nm). When the spinning voltage was 60 kV (Figure 4c), the nanofibers were mostly in the form of fiber bundles, the particle agglomeration was serious, and the average nanofiber diameter was the coarsest (563.08 ± 104.89 nm). This was because the higher the voltage, the greater the electric field force applied to the jet, and the more the jet was stretched, making the fiber diameter smaller. However, when the voltage was too high, the electric field force was too large, so that the spinning speed was too fast, and the jets were unstable and could not be fully stretched, resulting in an increase in fiber diameter and the easy formation of fiber bundles. This result is also consistent with that in Figure 2e–g. Furthermore, it could be clearly seen from Figure 4d that the agglomeration of nanoparticles in the fibers prepared by the SSFSE at 50 kV was very obvious (see the red circle), proving the advantages of EMAI.

**Figure 5:**
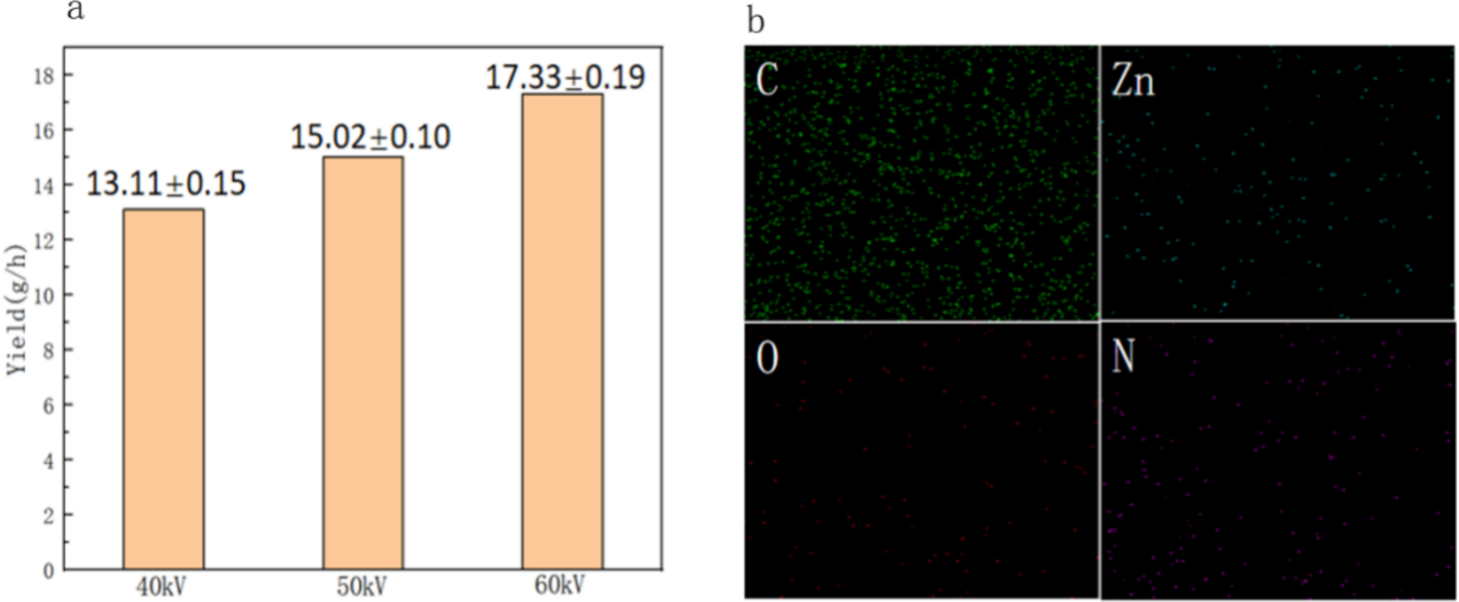
The yields of nanofibers obtained with different spinning voltages at the air flow rates of 50 m^3^/h (a) and EDS spectrum of nanofibers (b).

Figure 5a displayed the yields of ZnO/PAN nanofibers obtained with different spinning voltages at an air flow rate of 50 m^3^/h, illustrating that the higher the voltage, the higher the nanofiber yield, which further proved the force analysis in Figure 1a and the analysis results in Figure 2e–g. The EDS analysis result of nanofibers exhibited in Figure 4b showed that the sample contained four elements C, N, O and Zn, indicating that ZnO was successfully loaded on the PAN nanofibers. Zn was uniformly distributed, as shown in Figure 5b.

### Electric field simulation

**Figure 6:**
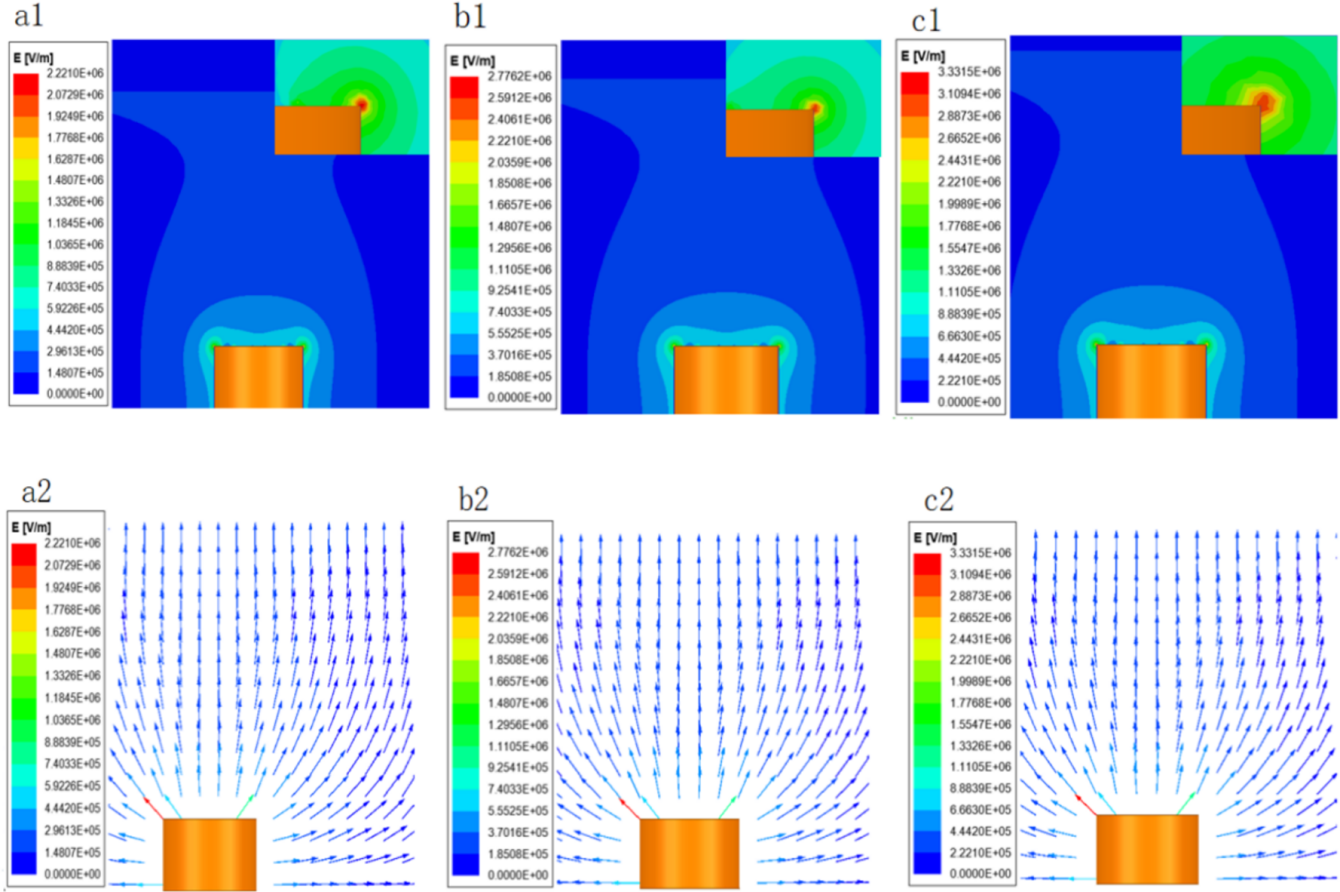
Electric field distribution at the porous spinneret at different voltages (40 kV (a), 50 kV (b), and 60 kV (c)).

Figure 6 shows the cloud diagrams (Figure 6a1, Figure 6b1, and Figure 6c1) and the corresponding vector diagrams (Figure 6a2, Figure 6b2, and Figure 6c2) of the electric field intensity near the porous spinneret under different voltages. It can be seen that the electric field intensity value at the uppermost edge of the porous spinneret was the largest. In the vertical and horizontal directions of the spinneret, the electric field intensity gets smaller farther away from the spinneret. According to the force analysis in point A in Figure 1a, when the electric field force (*P*_E_) acting on the spinning solution surface was greater than the solution surface tension (*P*_s_), unstable fluctuation on the free surface of solution easily occurs, thus significantly increasing the probability of multijet formation [25–26].

**Figure 7:**
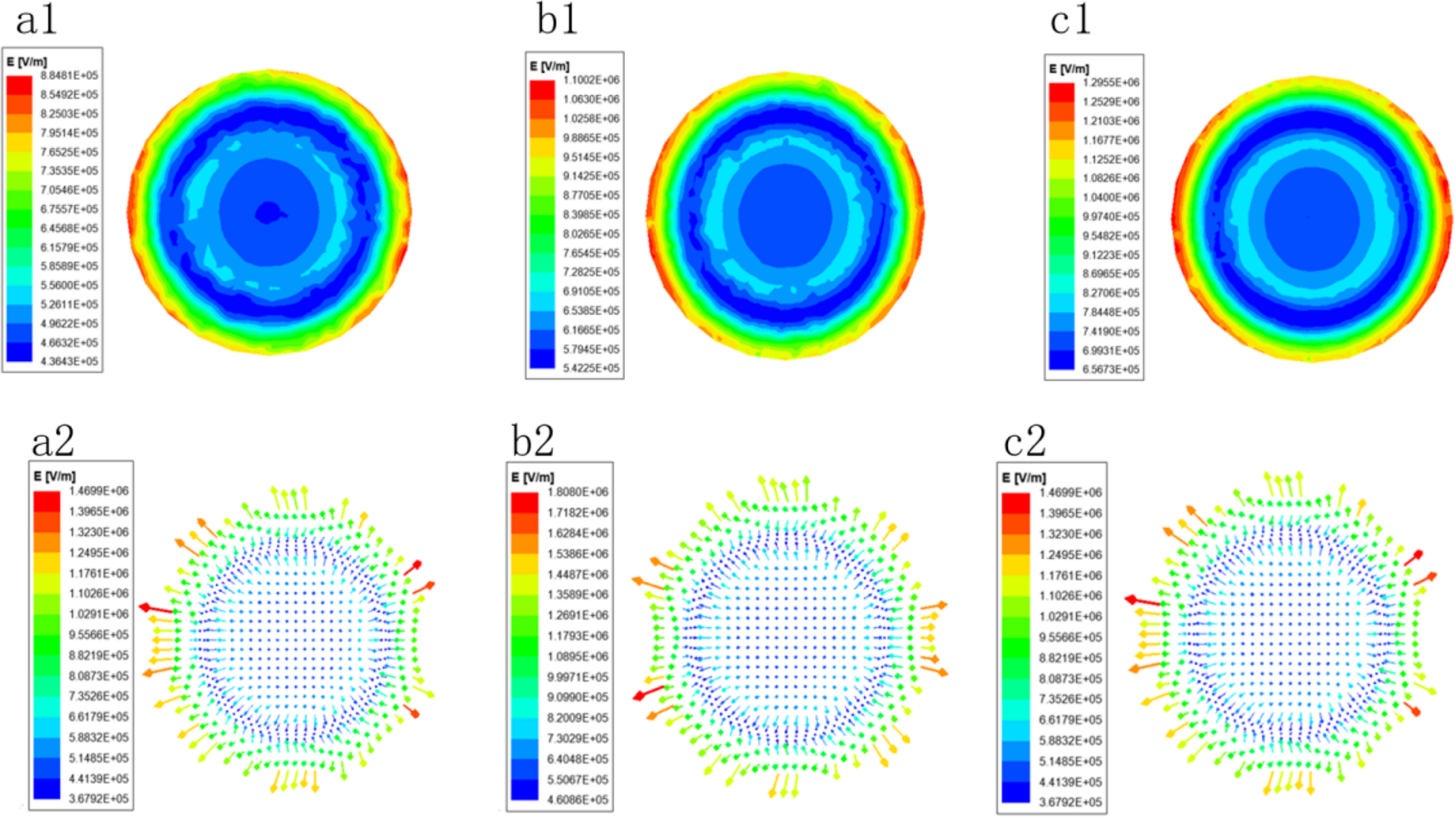
Distribution of electric field intensity at the top of spinneret under different voltages (40 kV (a), 50 kV (b), and 60 kV (c)).

**Figure 8:**
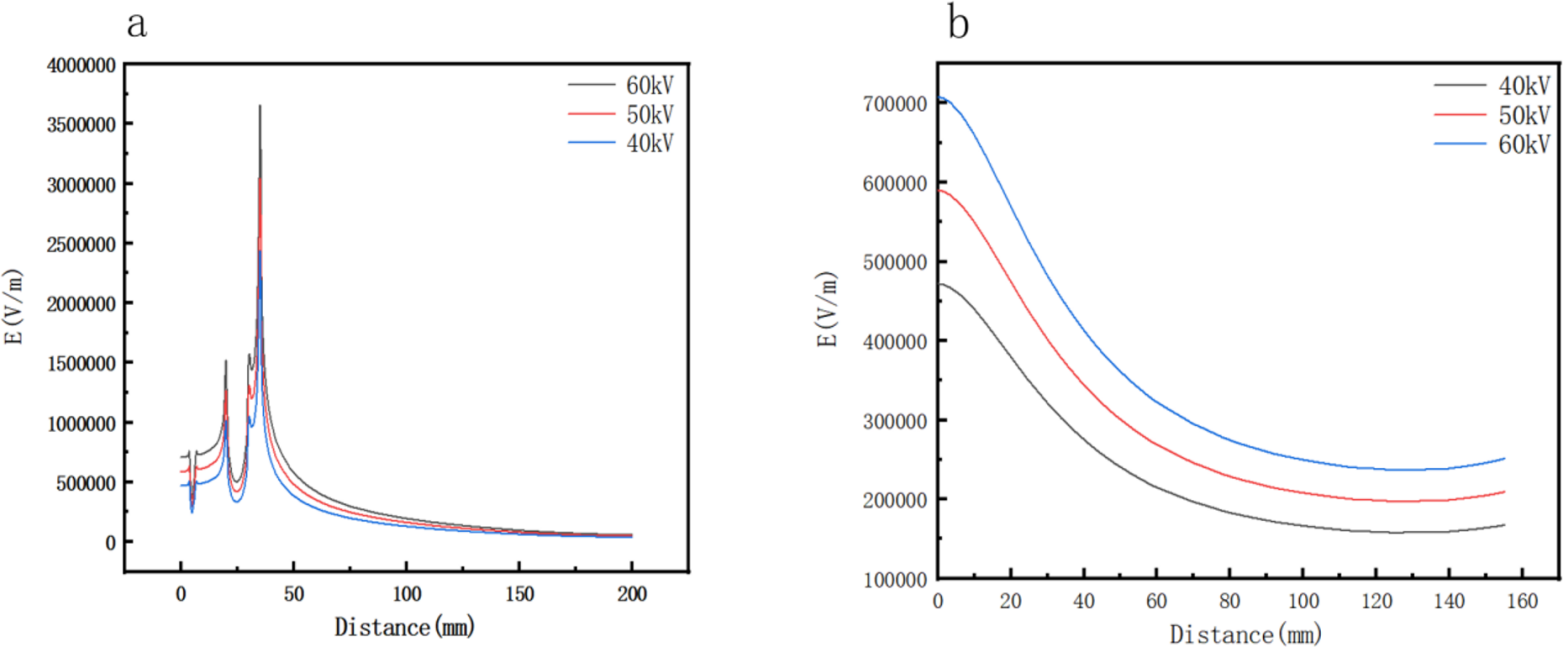
Radial (a) and axial (b) electric field distributions of the porous spinneret at different voltages.

In order to study further the electric field forces on the spinning solution surface under different voltages, the electric field intensity distributions on the surface were analyzed, as shown in Figure 7, which further proved the above conclusions obtained from Figure 6. The radial and axial electric field distributions of the porous spinneret under different voltages are illustrated in Figure 8. Figure 8a shows that in the radial direction, the electric field intensity dropped at 5 mm, which was caused by a round hole with a radius of 1 mm. At 20 mm, the electric field intensity increased greatly due to the boundary between the middle circular platform and the groove, where one side was a copper conductor and the other side was the solution, forming a large potential difference resulting in the increase of electric field intensity [27]. Similarly, the electric field intensity dropped sharply and afterwards increased sharply at the junction of the groove and the spinneret edge (30 mm). At the edge of the porous spinneret (30–35 mm), the electric field intensity was further improved greatly due to the tip effect [28], and the electric field intensity at the outermost edge of the spinneret reached the maximum value. This was consistent with the actual spinning situation, which also explained that most of the jets were produced on the edges and in the vicinity of the edges, as shown in Figure 2. Simultaneously, it could be seen from Figure 8b that in the axial direction the electric field intensity decreased continuously with the increase of spinning distance. The above simulation results explain the spinning effects of EMAI under different voltages, indicating that as the spinning voltage is increased, the number of jets generated on the spinning solution surface increases due to the increase of electric field intensity.

## Conclusion

An electrospinning device with multiple air inlets (EMAI) was used to prepare ZnO/PAN nanofibers in batches. It was found that the distribution of ZnO nanoparticles in the composite nanofibers obtained by EMAI was more uniform than that by SSFSE. This was because the air flow through multiple pores enabled the ZnO nanoparticles in the spinning solution to maintain uniform dispersion in the EMAI spinning process and reduced the agglomeration of nanoparticles. The influence of air flow rate on the spinning effect was investigated through experiments, which showed that too large or too small air flow rate was not conducive to obtaining high-quality functional nanofibers. When the air flow rate was 50 m^3^/h, only few microbubbles were generated, thus yielding ZnO/PAN nanofibers with uniform ZnO nanoparticle distribution.

In addition, the influence of the spinning voltage on the EMAI processes as well as the quality and yield of ZnO/PAN nanofibers were investigated experimentally and theoretically. The theoretical and experimental results were consistent and showed that as the spinning voltage increased, the number of jets generated on the spinning solution surface increased. However, an excessive spinning voltage (60 kV) would make the jets unstable and the spinning speed too fast, resulting in thicker fibers and fiber bundles. When the voltage was 50 kV, the spinning process was stable, and the quality and yield of ZnO/PAN nanofibers were high. This provides a convenient and effective method for batch preparation of functional nanofibers uniformly loaded with nanoparticles, thus expanding the practical application of functional nanofibers in the future.
